# Indigenization, Personalization, and Intersections: A Qualitative Study to Identify Essential Components for Developing an Indigenous-Centered Dementia Care Model in Alberta

**DOI:** 10.3390/ijerph23070883

**Published:** 2026-07-08

**Authors:** Jaiden Kuchinka, Richard T. Oster, Zahra Goodarzi, Zack Marshall, Lynn Jackson, Jayna Holroyd-Leduc, Vivian Ewa, Lisa Bourque Bearskin, Dallas Seitz, Jennifer D. Walker, Pamela Roach

**Affiliations:** 1 Department of Community Health Sciences, Cumming School of Medicine, University of Calgary, 3D10, 3280 Hospital Drive NW, Calgary, AB T2N 4Z6, Canada; jaiden.kuchinka@ucalgary.ca (J.K.); zahra.goodarzi@albertahealthservices.ca (Z.G.); zack.marshall@ucalgary.ca (Z.M.); jayna.holroyd-leduc@albertahealthservices.ca (J.H.-L.); 2 Indigenous Wellness Core, Alberta Health Services, 8440 112 Street NW, Edmonton, AB T6G 2B7, Canada; roster@ualberta.ca; 3 Department of Agricultural, Food & Nutritional Science, Faculty of Agricultural, Life, and Environmental Sciences, University of Alberta, 116 St and 85 Ave, Edmonton, AB T6G 2R3, Canada; 4 Department of Medicine, Cumming School of Medicine, University of Calgary, 3330 Hospital Dr NW, Calgary, AB T2N 4N1, Canada; 5 O’Brien Institute for Public Health, University of Calgary, Calgary, AB T2N 4Z6, Canada; 6 Hotchkiss Brain Institute, University of Calgary, Calgary, AB T2N 4N1, Canada; 7 School of Social Work, McGill University, Montreal, QC H3A 1B9, Canada; 8 University of British Columbia, 6200 University Blvd, Vancouver, BC V6T 1Z4, Canada; 9 Department of Family Medicine, Cumming School of Medicine, University of Calgary, 3330 Hospital Dr NW, Calgary, AB T2N 4N1, Canada; vivian.ewa@albertahealthservices.ca; 10 Beaver Lake Cree Nation, School of Nursing, University of Victoria, Victoria, BC V8W2Y2, Canada; bourquebearskin@uvic.ca; 11 Centre for Addiction and Mental Health, Toronto, ON M5S 3G9, Canada; dallas.seitz@camh.ca; 12 Department of Psychiatry, Temerty Faculty of Medicine, University of Toronto, Toronto, ON M5T 1R8, Canada; 13 Department of Health Research Methods, Evidence, and Impact, Faculty of Health Sciences, McMaster University, 1280 Main Street West, Hamilton, ON L8S 4L8, Canada; jennifer.walker@mcmaster.ca

**Keywords:** culturally grounded care, indigenous health, dementia care, health equity, indigenous methodologies

## Abstract

**Highlights:**

**Public health relevance—How does this work relate to a public health issue?**

- There is an expected increase in the number of Indigenous people living with dementia over the next 25 years. Enhanced care quality is imperative to improving Indigenous health outcomes.

**Public health significance—Why is this work of significance to public health?**

- Indigenous-specific dementia care is an urgent public health priority due to higher prevalence rates and specific systemic and structural factors in health systems and society.

**Public health implications—What are the key implications or messages for practitioners, policy makers and/or researchers in public health?**

- Indigenous-centered approaches to dementia care provision can improve quality of life for an aging population and increase availability of culturally congruent supports for families and communities.

**Abstract:**

Current models of dementia care often perpetuate the legacies of colonization, which highlights the need for a culturally congruent approach. This research describes the necessary components to co-develop a freely available Indigenous-centered dementia model of care in partnership with Indigenous individuals living with dementia, their families, and communities. To prioritize ethical and decolonial research approaches, sequential focus groups were used alongside Keeoukaywin (The Visiting Way)—an Indigenous methodology deeply rooted in Métis and Cree ways of knowing—guided by a group of advisors and Elders. The data were co-analyzed using reflexive thematic analysis guided by Indigenous principles. Three themes were generated: Indigenization, Personalization, and the Intersection of Challenges and Innovations, to illustrate how dementia care can be operationalized and adapted to various cultural and care contexts. This research offers a foundation for developing dementia care that truly aligns with Indigenous ways of doing.

## 1. Introduction

Dementia refers to a broad range of conditions of the brain that lead to progressive changes in memory, cognition, behavior, and the ability to carry out daily activities [1]. This condition is a growing global health concern, with current estimates indicating that 46.8 million people worldwide are living with dementia [2]. In Canada, it was estimated that in 2020, 10,800 Indigenous Peoples (First Nations, Métis, Inuit) were living with dementia [3]. By 2050, this number is expected to increase by 273% to 40,300, much higher than the 187% expected increase for the overall population [3]. In response to the historical and ongoing impact of colonization on health disparities, as explained by the Truth and Reconciliation Commission of Canada (TRCC) [4], this project addresses the significant gap in dementia care for Indigenous communities.

The need for healthcare services designed by and for Indigenous Peoples has been a priority for the last two decades (RCAP, 1996) and is outlined in health-related Call to Action #22 from the TRCC [4]. Care models suggested in the literature propose incorporating culturally congruent practices that resonate with Indigenous experiences and perspectives [5,6,7]. In practice, cultural congruency extends beyond cultural safety to ensure that care is fundamentally aligned with, and reflective of, cultural values, worldviews, and lived realities of the individual or community being served. This means that it is not only about respecting cultural differences but structuring care so that it is delivered through the lens of and individual’s culture. Research suggests that dementia assessments and care models should be developed in partnership with Indigenous people, promoting culturally congruent communication and addressing power imbalances within the health care system [5].

While there is variation in care models between Canadian provinces, Alberta has been one of the first provinces to engage in significant policy reform [8]. These policy changes focused on Alberta’s continuing care system to further expand the scope of care options offered [8]. Despite this prioritization of developing new care options, there remains a gap in care that is both relevant and safe for Indigenous Peoples [9], and guidelines are needed [10]. Current literature highlights the need for culturally congruent or responsive health care practices, yet there is a significant gap in the actualization of a care model that is both informed by and tailored to Indigenous experiences [5,9,11,12]. In response to this gap, Ody et al. developed a foundational conceptual framework for Indigenous-centered dementia care in Alberta through qualitative research [13]. This framework identified three interconnected domains: Relationality, Being Well, and Safety, as core principles of culturally congruent dementia care [13]. Guided by the methodology of Keeoukaywin (The Visiting Way) [14] alongside a community-based participatory approach [15,16], the present study builds on Ody et al.’s framework by exploring how these domains can be operationalized within care design and implementation across various care contexts in Alberta [13]. Preliminary results for this study were presented as a research poster at the Alzheimer’s Association International Conference, July 2025 and this manuscript provides the full findings with expanded data [17].

## 2. Materials and Methods

A community-based participatory research approach [15,16] was used with the research methodology, Keeoukaywin [14], to prioritize community engagement, relationality, mutual benefit, and shared authority over the research process [18]. Community-based participatory research [15,16] offered tangible practices for community engagement, while Keeoukaywin [14] provided the underlying values, worldviews, and cultural integrity, guiding how collaboration occurred. Keeoukaywin points to special times and spaces in which connections are strengthened; individuals are reminded of who they are and their responsibility for the well-being of the whole. The focus on relationality, communal learning, and shared experiences broadened the idea of what research methodology can look like. This resulted in a robust methodology that corrects inaccuracies in how Indigenous ways of being, knowing, and doing are understood, particularly the misconception that stories are anecdotal, subjective, or less rigorous forms of evidence, in addition to eliminating the narrative that all Indigenous Peoples are the same.

### 2.1. Indigenous Engagement

This work was done together with a group of advisors consisting of Elders, people living with dementia and care partners, Indigenous community members, and health care providers from local Indigenous Nations and organizations. The advisory group was engaged throughout the entire research process from conceptualization and data collection, to analysis and knowledge sharing. We also engaged collaboratively with Indigenous health and social care organizations and Indigenous communities during the conceptualization of the study to ensure we were answering questions that are relevant and important to Indigenous communities and individuals. This collaboration is based on long-standing relationships between the senior author (PR) and all partners provided letters of support. The advisory group was updated regularly and provided feedback on all methods and preliminary results and analysis.

The research team is made up of Indigenous (JK, PR, LBB, JW, LJ) and non-Indigenous researchers (RTO, ZG, VE, JHL, DS, ZM) that bring a diversity of lived experiences and identities. The first author (JK) locates herself as a woman with Austrian, Ukrainian, and Saulteaux ancestry with her paternal side of the family coming from Fishing Lake First Nation and Yellow Quill First Nation. The senior author and project lead (PR) is a citizen of the Métis Nation within Alberta and originally comes from the Métis community of St. Laurent Manitoba. She has been working in research with families living with dementia for 20 years and works closely in relationship with the Métis Nation within Alberta to advance research with meaningful impact to community.

### 2.2. Sampling and Recruitment

This study included participants who self-identified as Indigenous (First Nations, Métis, or Inuit) residing in Alberta and being a person living with dementia, a family member or care partner of a person living with dementia, or a community member with other experience with dementia. Convenience sampling [19] was used to recruit from the pool of eligible participants who had consented to further contact from previous research engagement, with recruitment occurring via email. Though this may have introduced potential social desirability bias, the participants all confirmed they felt comfortable to speak candidly about their experiences with one another. The participants did not have a prior relationship with the first author, who conducted all of the data collection, and this helped mitigate any further potential impact of social desirability bias. Five participants capable of offering in-depth insights and thick description [20] into culturally congruent dementia care were recruited. The sample size was justified by a combination of pragmatic, ethical, and methodological considerations. These intersecting factors included the breadth of the research question, the anticipated richness of data, the identity and experiential diversity of participants, and the practical and ethical constraints of the project. This study employed four sequential focus groups conducted over four months; this format provided both depth and continuity, fostering the ability to capture richer understandings than a higher number of shorter, one-time interviews would have been able to. Though the number of individual participants may be considered low, the depth of the data collected over recurring meetings was rigorous due to the nature of the sequential focus groups. The sample size was not only justified on ethical and practical grounds due to the sensitive nature of discussing experiences of dementia care but also ensured there was adequate space for everyone to share their experiences [21,22]. Participants were selected based on their diverse experiences with dementia, including early experiences of memory loss, as disclosed by the participant, and the presence of different care partner arrangements. All participants in this study were English-speaking. Capacity to consent was assessed through dialogue and relational engagement rather than clinical evaluation, recognizing that the ability to reflect on and share one’s experience differs in its standard of decision making than making medical or treatment related decisions. For participants living with dementia, consent was continually revisited using process consent [23] by ensuring willingness and comfort through gentle questions and observation. The research team, experienced in working with people living with dementia, prioritized respect for a participant’s self-identified capacity and viewed that it would be unethical to exclude individuals based on their diagnosis alone. Participants were reminded that if at any point throughout data collection they felt distressed or wished to pause or stop that they were able to do so for any reason. Participants were reminded that they could pause or withdraw at any point, without impacting their honorarium, ensuring that consent remained informed, relational, and an ongoing process. No participants requested a pause or withdrawal, and no instances of distress requiring intervention occurred during the focus groups. However, participants shared appreciation of having these options available as it supported their sense of agency.

### 2.3. Data Collection

Sequential focus groups [22] were used for the data collection process based on the goals to foster trust and build rapport, provide space/time for each participant to reflect on their experiences and share them in a safe manner, give space to each participant in between conversations to reflect on the focus groups, and understand the in-depth and complex experiences of each individual. Data collection occurred from October 2024–December 2024. This sequential design allowed for deep insight into the topic and was further established over time with each focus group building from the previous conversation. The semi-structured sequential focus group guides were drafted by the first author (JK) informed by research from Ody et al. [13], and further developed in consultation with the advisory group based on emailed feedback of the full guides. Ody’s (2026) framework served as a guiding structure for both the development of focus group questions and the analysis process [13]. The first three sequential focus groups were designed to explore one of the framework domains of Safety, Being Well, and Relationality in the context of dementia care, with the final focus group concentrating on specific care delivery design and implementation [13]. This ensured that the study remained grounded in Indigenous experiences of dementia and shaped the thematic analysis by linking the emergent themes to the broader relational and cultural contexts they arose from. Sample questions included: “How can cultural practices and traditions be incorporated into dementia care to strengthen relational ties within the community?” (FG1), and “How can we ensure that health care workers are practicing culturally congruent care?” (FG3), and “What types of activities should be included to engage participants and meet the goals of Indigenous-centered dementia care based on our previous discussions?” (FG4). The focus groups were facilitated by the first author (JK) and guided by Keeoukaywin [14]. In practice, this meant ensuring the authors understood their positionality and relation to the participants and data. The first author’s insider position as an Indigenous person may have supported relational comfort within the focus groups, which may have facilitated participants’ comfort in sharing their experiences. Relationality was essential to the data collection, with the first focus group being in person with a shared meal. The following three focus groups occurred online at the request of participants due to the risks of travel during winter months. This mixed modality supported safety, relationality, and participant preference. Research contexts inherently involve power dynamics through institutional affiliation, facilitation roles, and control over research processes, and attention was given to how these dynamics can shape engagement and knowledge production. The participants were not merely seen as research subjects with information to be extracted, but as humans with unique stories and lives. To ensure every participant had the opportunity to speak, the facilitator ensured the next question was not asked until all participants had the space to share as much as they wanted to in response to the initial question. This approach supported meaningful participation from all participants, including those living with dementia. Field notes were recorded during and after the sequential focus groups that ensured the information gathered captured a comprehensive record and to support triangulation of data [20]. These field notes were reviewed alongside the transcripts during data immersion and analysis, providing further context and informed the development and refinement of codes and themes. These were further informed by the literature and previous research led by the senior author (PR), ensuring that the model is not only relevant but also adaptable for other communities and nations. The sequential focus groups were recorded via an audio recorder and transcribed verbatim. The recordings were transcribed by a professional, ethics-approved transcription service (Rev.com) and verified for accuracy by reading the transcript while listening to the audio.

### 2.4. Data Analysis

NVivo (Version 14) software was utilized to manage the data. The data were co-analyzed using reflexive thematic analysis [24] guided by Indigenous principles of contextual reflection, relational accountability, and prioritization of Indigenous ways of knowing [25]. Analysis was completed as an extension of the relational and holistic principles established during data collection. Consistent with Keeoukaywin, each code was understood as representing a participant’s lived story rather than a data point [14]. Throughout analysis, coding and thematic construction were made with ongoing awareness of the researcher’s positionality and relational responsibility to each participant. Analysis followed Braun and Clarke’s six guiding phases of reflexive thematic analysis: (1) familiarization with the data, (2) generating initial codes, (3) constructing themes, (4) reviewing potential themes, (5) defining and naming themes, and (6) producing the analytic report [21]. This process was anchored in the teachings of Keeooukaywin, with deep reflection of the relationship with the data itself [14]. Data familiarization involved multiple readings of the complete dataset and immersion in the transcripts. This process supported attention to pauses, tone, and conversational dynamics that may not have been fully captured during facilitation but emerged through careful transcript review. Given the relational practice guided by Keeoukaywin, data saturation was determined not by conventional saturation practices of information redundancy, but by the relational depth that was an outcome of the sequential focus group design [21]. Western standards of data saturation often sit in tension with Keeoukaywin, where knowledge is not extracted from a large number of participants but co-produced through consistent relationship over time [14]. Participants returned across four focus groups, each building on the prior conversation so that the design produced a cumulative relational depth rather than a thematic accumulation. This is consistent with Gaudet’s [14] framing of Keeoukaywin as a living practice of coming to know, where knowledge is enriched through repeated visiting rather than bounded in repetition. Line-by-line coding was conducted to generate initial descriptive codes by JK. An audit trail and memos were used to document notes, additions, removals, or code merges throughout the analytic process. Following the initial round of coding, the dataset was re-coded using the developed codebook to ensure consistency and comprehensiveness across transcripts. To enhance analytic rigor and confirmability, coded transcripts and the evolving codebook were reviewed by the senior author (PR), who conducted a secondary review to identify potential gaps or alternative interpretations. Following coding, related codes were grouped and organized into themes through the development of multiple thematic maps. These maps supported critical reflection on the analytic structure, including consideration of thematic coherence, boundaries, and relevance to the research aim. Through iterative peer debriefing with the research team and advisory group [26], a preliminary thematic framework identifying components required for an Indigenous-centered approach to dementia care was developed. This involved ongoing discussion among Indigenous and non-Indigenous members of the research team, grounded in trust and long-standing relationships. Interpretation was guided by the Indigenous researchers leading the work to ensure the findings were grounded in Indigenous ways of knowing. While Ody et al.’s framework was used as a sensitizing lens during analysis, it did not constrain coding or theme development. Inductive codes emerging from participants’ narratives were used to expand the framework rather than recreating it. Participant quotes were edited minimally for readability, including minor grammatical corrections and removal of filler words, while preserving meaning and context. Once the thematic framework was drafted, it was shared with participants as a form of member checking; however, individual quotes were not reviewed separately [26]. Once the participants expressed alignment, the framework was reviewed by the advisory group to further strengthen credibility and research aims. The advisory group provided feedback via discussion until a consensus was reached to finalize the thematic framework.

### 2.5. Ethical Considerations

All authors have completed the Tri-Council Policy Statement: Ethical Conduct for Research Involving Humans (TCPS 2) and are registered members of the Institutional Research Information Services Solution (IRISS). This study was approved by the Conjoint Health Research Ethics Board at the University of Calgary (REB22-1413). Due to the diversity within urban Indigenous populations, this research was guided by several research ethics frameworks: the First Nations Principles of Ownership, Control, Access, and Possession (OCAP^®^) (OCAP^®^ is a registered trademark of the First Nations Information Governance Centre), the Principles of Ethical Métis Research by the National Aboriginal Health Organization [27], and the National Inuit Strategy on Research [28]. Although the focus group guide (see Supplementary Materials) did not explicitly seek disclosures of traumatic experiences, the nature of the discussions often brought difficult memories to the surface. To support participants’ emotional, spiritual, mental, and physical wellbeing, each session began with a smudge, and participants were reminded they could pause, skip questions, or withdraw from the study at any time. At the end of each session, the facilitator (JK) shared self-care strategies and reminders for the participants.

### 2.6. Rigor

Several principles of rigor were applied throughout the research process: (1) Transparency, (2) Validity, (3) Reliability, and (4) Reflexivity [20]. Transparency was established by stating the positionality of each author, clearly outlining the analytic procedures used, including the coding strategy and theme development, and creating an audit trail within NVivo. Specifically, this audit trail documented the rationale for each code, the basis for any merges or removals, and the justification for thematic development. Validity was practiced through member checking, where a draft of the preliminary analysis was emailed to the participants to ensure it was representative of their vision, experiences, and thoughts, and following the First Nations Principles of Ownership, Control, Access, and Possession (OCAP^®^), the Principles of Ethical Métis Research by the National Aboriginal Health Organization [27], and the National Inuit Strategy on Research [28]. Participants had the option to provide feedback verbally or by email, where they expressed their experiences and thoughts were represented, and the draft was then shared with the advisory group, aligning with the relational accountability described in Keeoukaywin [14]. Reliability was ensured through recording and verbatim transcriptions of all the focus groups [20]. Additionally, the transcripts were read multiple times to ensure the codes and themes were an accurate representation of the dataset. Secondary coding was conducted by the senior author (PR) that allowed cross-checking to enhance the reliability of the findings [20].

## 3. Results

Participant demographics are presented in Table 1 using pseudonyms for each participant to protect their identities. Five participants were recruited into the study and took part in all four sequential focus groups. The sequential focus group schedule, outlined in Table 2, began on 6 October 2024, and was completed on 16 December 2024. Participants described distinct experiences with dementia care, including roles as care partners and professional experience within dementia care settings. The participant ages ranged from 36 to 67, with a median age of 56 years. Most participants were women (*n* = 4) with one man participating.

**Figure 1:**
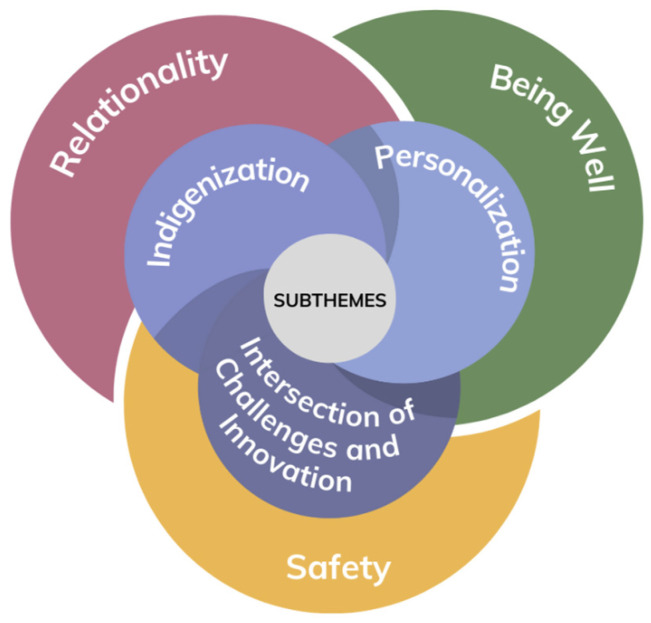
Thematic Framework and Relationships.

Three themes were generated to understand what is needed to create an Indigenous-centered dementia care model that respects the diverse identities of Indigenous people in Alberta: Indigenization; Personalization; and Intersection of Challenges and Innovations. These themes function as implementation centered insights derived from participant experiences. Ody et al.’s framework [13] (Relationality, Being Well, and Safety) informed the interpretation and development of the themes, with the relationship between the framework and themes illustrated in Figure 1. The thematic map presented in Figure 1 underwent multiple iterations throughout the analysis. Initial versions were drawn by hand to map relationships and categories from the codes. The subthemes (see Table 3 middle column for subthemes) are positioned at the center reflecting how they emerge through the interaction of the framework and themes.

These were then reorganized and refined through peer debriefing, member checking, and ongoing discussions among the research team to ensure conceptual coherence with the study objectives. Subthemes, shown in Table 3, provide further detail on how participants described current care experiences and the changes they viewed as important for safer, culturally congruent dementia care.

### 3.1. Theme 1: Indigenization

Participants described Indigenization as encompassing culturally congruent needs and practices, including dementia-friendly ceremonial opportunities, cultural care planning, and supportive physical spaces (e.g., cultural rooms). Across discussions, participants framed Indigenization as requiring more than the presence of cultural elements but rather focused on the conditions that make cultural practices possible in care settings, including staff knowledge, protocol, and environmental accommodation.

#### 3.1.1. Subtheme: Family Connection

Participants emphasized that family was central to well-being and safety in dementia care. Family was discussed as fluid and expansive, extending beyond nuclear family to include extended kin, community members, and ancestors. Overall, participants described family connections as a foundational expectation in care, with implications for how programs and staff roles are organized. This was reflected by Mary and Joseph who described the importance of the inclusion of community in care, referring to staff as a community for patients, not just care partners.

*“So, you know, it’s not foolproof, but caregivers have to have support groups, and it must be community-based… That’s what the Indigenous population is all about, it’s community-based caring. So, I think in the future, that’s where it has to go for our Indigenous loved ones. It’s not just the staff; it’s a community of people.”*

—*Mary, FG3*

*“She almost became childlike for a period of time. You know, she just didn’t quite get it. But my sister wanted to make sure that she got everything on the table for her, when my mom wasn’t in capacity to deal with it. And understanding that the person who’s going through that has, they’re having their own journey.”*

—*Joseph, FG1*

#### 3.1.2. Subtheme: Connection to Culture

Participants described creating space for those living with dementia and their care partners to connect to their culture, in whatever way they choose, as crucial. Specifically, participants mentioned the call for physical spaces to practice cultural traditions and the importance of cultural teachings for both individuals living with dementia and their care partners was stressed. There was also a need for individual connection to culture, and a need to not pan-Indigenize culture. Mary and Dorothy expressed these views:

*“There should be teachings, Elders must be a part of that because that’s who the third person is. You need an Elder to come in, and understand, and talk so that everyone is in a circle for that one person in the middle.”*

—*Mary, FG3*

*“Some of the questions need to be way more specific too, because even when I’m asked, ‘Do you practice your culture?’ What does that mean? Like, what part of culture are you talking about? It could be just about anything… but those questions need to be way narrowed down. Do you speak Cree? Do you understand Cree? Do you practice smudging? Like that. Not as a big umbrella to say, ‘Do you practice your culture?’ One day I started saying, ‘I am culture, I’m Indigenous.’”*

—*Dorothy, FG4*

#### 3.1.3. Subtheme: Understanding the Context

When linking dementia care models to the contexts of individuals and groups, this needs to be thought of both individually and collectively. Having further context to each person’s story, where they come from, and their history and current realities was shared as something crucial to providing a care model for Indigenous populations in Canada. This also includes understanding the distinct differences between Indigenous populations, communities and Nations across Canada.

*“…And so, when you say culturally sensitive, it’s to ask those questions of, “Okay, you know, do I have any preconceived notions of who this person is? How can I remove those preconceived notions and just ask who they are, where they’re at, and how to approach where they’re at?”… Everyone has presumptions. And removing those presumptions is what allows for cultural safety.”*

—*Joseph, FG3*

#### 3.1.4. Subtheme: Connecting Back with the Land

Participants described land and nature as important for well-being and orientation, and framed access to outdoor space as a practical and meaningful part of care. Alice described how remaining indoors can affect people living with dementia. Connecting back to the land through dementia progression can be done in various ways, with participants coming up with various ideas such as berry picking, daily walks, and bird watching/feeding. Alice further mentioned the need for the feeling of having purpose through these outside activities when connecting to the land.

*“I was thinking with the daily walks, like, even if it was just to get outside and feed birds. I know a lot of patients are really interested (laughs) in checking out the birds every day. Because… I know that they do want to have a purpose. They don’t want to just go stand out in the cold. Sometimes in Alberta it could be freezing. But if they had, like, birdseed or something.”*

—*Alice FG2*

#### 3.1.5. Subtheme: Shared Experiences

Participants described shared experiences as important for relationality among individuals living with dementia, care partners, staff, and community. Mary described how physical space design (e.g., circles and round tables) can support community-oriented communication.

*“I think some of the physical adaptations need to be focused on that community. Um, like round tables, there should always be round tables and there should be sitting circles. I mean, the circle is a part of the culture of learning, you know, the culture of communication.”*

—*Mary, FG3*

### 3.2. Theme 2: Personalization

Personalization reflects how participants described the need to customize and adapt care, culture, and daily routines to each person’s identity, preferences, and changing needs. Personalization, as discussed by participants, is not about assuming what care should look like just because someone is Indigenous, but rather asking, listening, and adjusting care in a way that honors the person at every step in their journey.

#### 3.2.1. Subtheme: Connection to Oneself

When experiencing symptoms of dementia, having opportunities to connect back to oneself, whether that be through hobbies, music, or visual cues like photo albums/pictures was stressed by participants. These simple interventions help anchor individuals in their own stories and give them the opportunity to maintain a sense of self even when cognitive abilities are changing.

*“Having family time and if family can’t be there that day, having photo albums or pictures. Um, and music or just having… I think that the feeling of their apartment or their room or wherever they are, seeing things that are very tangible, because I think visual cues are what they rely on, right?”*

—*Mary, FG2*

#### 3.2.2. Subtheme: Reframing Well-Being

Participants shared their perspectives on what it means to be well when living with dementia. Alice described well-being more broadly, as being all parts of the medicine wheel: Mental, Spiritual, Emotional, and Physical. They highlighted the importance of creating opportunities for those living with dementia to feel well in all these areas.

*“Maintaining your well-being is all parts of the medicine wheel, and I feel like there are people who can come in, right, like, if your family can’t, or just to support the family with the patient in doing a ceremony. They can even just be a social visit, like nice to see people of the same culture in the room.”*

—*Alice, FG2*

The importance of humour when thinking about well-being was noted by all participants. While this was described as a trauma response, it was also described as being part of Indigenous culture.

*“Humour is so important. So, we’re… you’re not insulting someone when you’re ribbing them. Humour is a trauma response. It’s so ingrained in Indigenous culture.”*

—*Joseph, FG1*

#### 3.2.3. Subtheme: Evolving Accommodations

The group emphasized that care needs shift over time and described routines and accommodations as important to safety. Alice noted the importance of routines, and how they may change as dementia progresses. Joseph simply summarized these descriptors with this short statement:

*“I think routines are really important. That, in their day-to-day that allow them to feel safe and they kind of have an expectation of what the day will bring. So, it might just be a slight change in their routine as the disease progresses.”*

—*Alice, FG2*

*“It changes…There’s no one size for all”*

—*Joseph, FG1*

#### 3.2.4. Subtheme: Autonomy

To further establish care that is personalized for each individual living with dementia and their families, autonomy was highlighted as a priority by the participants. Alice described how rigid mealtime routines can feel institutional and misaligned with individual preferences and how individuals are just expected to fit in with the dominant culture, which may not be culturally congruent.

*“Yeah, it’s super regimented, right? Like, some people sleep in and then they miss breakfast. Or some people have never in their life had breakfast, and now they have to have it (laughs). It’s just not honoring each person, right? Like, it’s just institutional.”*

—*Alice, FG2*

#### 3.2.5. Subtheme: Diverse Education Opportunities

To ensure that the personalization of care can be effectively delivered in care settings, participants expressed the need for diverse education opportunities for care staff, care partners, family members, and those living with dementia. Participants highlighted the importance of networking and creating connections to other organizations and services. They also wanted interactive and flexible supports and education for both formal and informal care partners. When discussing what educational opportunities should look like, one participant commented on the importance of centering on those with lived experience as a source of education.

*“People who have lived experiences should be able to use their voice to educate.”*

—*Dorothy, FG3*

### 3.3. Theme 3: Intersection of Challenges and Innovations

Participants described care system features that can slow, prevent, or limit the ability to deliver effective and safe dementia care, and shared strategies they viewed as promising for improving care. This theme captures how structural barriers and harms intersect with participant-generated ideas about accountability and support strategies.

#### 3.3.1. Subtheme: Institutional Reminders

Throughout the focus groups, participants described institutional reminders in dementia care settings that evoked association with residential schools, including the design and power dynamics. Participants shared how care environments can trigger these memories. These reminders may further compound as individuals progress in their dementia journey. Participants connected these experiences to the importance of trauma-informed environments that account for emotional and cultural harm, not only physical safety. This was reflected by Delores, who noted her husband had more memories as the symptoms progressed. His willingness to speak of them was rare, reflecting how deeply rooted these harms can be.

*“My husband also was in residential school for many years. And, as he got sicker, of course, he had more memories of his time there. He didn’t speak of residential school very often during his life, nor to very many people. I was probably one of the very few. But he, um, would think of the, the olden days.”*

—*Delores, FG3*

#### 3.3.2. Subtheme: Barriers to Engage

Participants shared concerns about the barriers to implementing culturally congruent care due to the constraints within existing care settings. One example of this was over the current food restrictions, as many facilities do not allow traditional meats because they do not meet the quality grade of meats from their typical food providers.

*“You can’t, you can’t take moose meat into a facility ‘cause it’s not, um, graded, quality meat or something.”*

—*Dorothy, FG1*

The lack of time and capacity among staff was a major concern by participants in terms of implementing a new care model. Participants’ accounts suggested that cultural inclusion alone was experienced as insufficient without addressing trust and relational approaches in everyday care.

*“There were not enough staff. They didn’t give a crap. It was to the point that I felt it was dangerous. I went and reported. … And, it was one of the hardest things that I have ever did was to struggle to find care for my husband that was appropriate. And it wasn’t easy. They wouldn’t let us bring food. We couldn’t have food in.”*

—*Delores, FG3*

Joseph shared the extreme difficulties of accessing cultural teachings and ceremony at a later stage in life if they are separated from their community.

*“She wanted to come to this stuff at a late stage in her life. So, having access to Elders and knowledge keepers and just that kind of stuff was very important to her…And there was no, readily, like, here’s a list of a local Elder, or anything. We had to do a lot of our own work, obviously.”*

—*Joseph, FG2*

#### 3.3.3. Subtheme: Unsafe Care Stemming from Racism

Experiences of unsafe care were shared among the group. This was reflected by participants who all had unique but equally harmful and unsafe experiences with family members seeking care. Participants shared experiences about the lack of effective and appropriate care, which was attributed to the colour of one’s skin and their Indigenous identity. Across each of the participants’ experience, they frame racism as directly shaping the current safety and willingness to engage with care systems. This experience reflects the direct racism that is felt by those seeking to access health care in an urban hospital in Alberta.

**Content warning**: Please read these participant quotes with care as they may be triggering.

*“I felt that because we were in a [city] hospital, that was supposedly well known for its care, they treated us like crap because he was First Nations, and I was Métis. And it was obvious that that’s how we looked with our skin. And we didn’t have enough Indigenous social workers to help us out. No one offered anything.”*

—*Delores, FG3*

*“And, the family didn’t know, but my cousin saw the nurse put something in his IV and she asked them, “What did you do to him? What did you give him?” They gave him Narcan because they thought he was high or he took something. But, you know, first stages of the dementia.”*

—*Dorothy, FG3*

#### 3.3.4. Subtheme: Accountability in Implementation

The importance of accountability when adopting a new care model to various settings was discussed. Each participant emphasized that trust in care environments depends not only on good intentions but on systems that offer transparency for families and communities to then assess and influence the quality of care. Immersive training was identified as an important feature of improving Indigenous-centered dementia care and should not be just an educational tool but a meaningful practice in relationality, cultural humility, and collective change. Throughout these conversations, accountability emerged as a layered responsibility between institutions, staff, families, and communities.

*“What about an immersive cultural training. Either a weeklong, or a weekend. I feel like the experience would be better for learning vs just reading. Like “actively working through the manual” vs reading through the manual. Could biannual or quarterly. I feel like it would also be a great retention and team building for current staff.”*

—*Alice, FG4*

*“So, with that, wouldn’t that be somewhere we could also look at starting? Is with the accreditation process and what those standards and those, and the gold standards or the lack of standards? Like, there needs to be education somewhere at that level with the higher-ups especially, about including, um, cultural safety.”*

—*Delores FG4*

Participants stressed that accountability should not end once staff complete one-time training. There needs to be a commitment to ongoing, reflexive learning that evolves alongside community needs and cultural contexts.

*“A lot of it’s continuing education. It can’t just be a single session, it can’t be something that, you know, you check mark the box and you’re done.”*

—*Joseph, FG4*

#### 3.3.5. Subtheme: Mobilizing Support

To ensure successful implementation of a culturally congruent care model, participants noted the importance of seeking out what is already available. Additionally, in leveraging current ones, there is a possible need for new supports. Creating a sense of understanding and community, both in care settings and in broader support networks, was seen as essential to counteracting isolation and the difficulties of diagnosis. Participants described mobilizing support as not just about increasing resources but supporting families to feel equipped and empowered to utilize them. Participants spoke about the social and emotional dimensions of support. Joseph described stigma and isolation as barriers and emphasized the importance of community-based support and speaking out.

*“Knowing that you can invite the others into the care to create that community, ‘cause it’s naturally isolating… and sometimes there’s a shame associated with it…the open dialogue of saying, “This person’s gonna need help, and I might need help, helping them.” You know, it’s very difficult, there’s a stigma around it.”*

—*Joseph, FG1*

*“And lots of times when you’re first admitted, you don’t have a lot to say ‘cause you don’t know what’s going to happen. And maybe if there could be a bit better of an orientation instead of just taken to the room shown where the closet is, shown where the bed is for their family members. Instead of just giving a handbook to people, that there could be an Indigenous person there to say, “You’re allowed to do this. Can they do smudges? Can they do this? Can they have bannock? Can they have…” Those, those kinds of simple, so simple but so important things to our family members.”*

—*Delores, FG3*

## 4. Discussion

This study identified three interconnected themes that are essential to consider when co-developing an Indigenous-centered dementia care model: Indigenization, Personalization, and the Intersection of Challenges and Innovations. Indigenization moves beyond tokenistic gestures or surface-level inclusion to critically address power imbalances to ensure Indigenous ways of knowing and doing are recognized and perceived as equal to western ways of knowing and doing. Participants stressed the need to ground dementia care in the diverse cultural practices of Indigenous communities, teachings from Elders, land-based activities, and family and community connections. They also emphasized the need for care that reflects each person’s unique identity, preferences, culture, and evolving needs over the course of their diagnosis. These findings suggest that culturally congruent dementia care cannot be achieved through the mere addition of cultural activities, but rather requires structural changes to how care environments, staff education, and daily routines are organized.

A 2024 scoping review explored existing models and outcomes for culturally safe dementia care interventions for Indigenous populations but found no studies that met the inclusion criteria [9]. It is important to note that this is not just a glaring gap in the literature; it reflects the critical absence of documented, evaluated, and culturally congruent dementia care interventions. This points to a likely scenario of dementia care systems not designed to respect and integrate Indigenous cultural, spiritual, and relational needs and preferences.

This gap in care sits within a broader landscape of inequities in Canadian health care. Historical and ongoing colonization has fostered cultural insensitivity, explicit and implicit interpersonal discrimination, and limited access to appropriate services—all contributing to health disparities [12,29]. Research in Alberta has shown the presence of explicit and implicit anti-Indigenous bias among physicians [30], reinforcing how racism can shape clinical decision-making and trust. Participants’ experiences in this study made these dynamics visible in everyday interactions. These are not isolated events, but patterned harm rooted in historical and contemporary colonial structures [12,29,30]. Furthermore, these experiences show the tangible dangers that can arise when there is a lack of specific brain-health and cultural education and accountability for healthcare providers.

Participants’ descriptions of care needs align with but also extend beyond western person-centered care approaches. Participants in this study emphasized that care must be understood through an Indigenous lens that reflects holistic conceptions of well-being, relationality, and connection to culture and land. At the same time, there has been an uptake in the implementation of non-pharmacological interventions in western dementia care models such as cognitive therapy, physical activity programs, sensory therapy, reminiscence therapy, and social engagement activities [31]. While these non-pharmacological interventions are aimed at maintaining cognitive function and overall well-being, there is a lack of evidence regarding cultural congruency and the applicability to Indigenous groups in Canada [5]. Therefore, Indigenizing dementia care requires more than adding activities; it requires shifting underlying cultural assumptions of care.

To reflect the reality of what a culturally congruent dementia care model could look like, this study is grounded in the voices and experiences of each participant, while also accounting for current care models. The insights align with broader policy recommendations, such as those outlined in *Honouring Our Roots* [32], a report developed by Indigenous Primary Health Care Advisory Panelists in Alberta, which calls for embedding cultural safety, Indigenous knowledge, and trauma-informed approaches across health systems. This aligns with the reflections, conversations, and experiences of each participant and their desired outcomes. The future of dementia care should center family and community because that is where care already happens, and where culture can be sustained more readily [6]. This requires working alongside communities to ensure they feel equipped to care for their loved ones in place. However, when residential care becomes necessary, community connection and culturally congruent care are essential, where participants called for traditional foods, language, prayer, and ceremony to be normalized rather than exceptional [6,33]. The Indigenization of a dementia care model is not just about including cultural practices here and there but about ensuring these practices and unique needs are readily accessible throughout every dementia journey and supported with compassion within every care setting.

Participants offered tangible innovative strategies for making culturally congruent care more feasible in practice. These included embedding culture, land, and identity into everyday life through ceremony (smudging, sweats, storytelling, dancing, pipe ceremony) delivered in dementia-friendly ways across various stages of someone’s condition. Language classes, access to nature, dedicated cultural rooms, personalized rooms, holistic strength-based intake and cultural charting (preferred practices, traditional food preferences, and pre-diagnosis hobbies/interests) were also identified as important by participants. Weekly calendars were envisioned to include optional programs like Elders’ storytelling, beading, land-based activities, traditional dancing, and transportation to local nation events such as powwows. Connection to family, community, and the land was consistently identified as a central priority for participants. This aligns with literature that frames culturally congruent care as a relational practice that integrates Indigenous knowledge and healing into care models [34].

The shift towards cultural congruency requires systemic commitments by care staff, government, and policy makers to address current power imbalances, create accountability measures, and mechanisms for continuous feedback and adaptation.

For real change to be achieved for Indigenous people living with dementia and their families and communities, systems must reduce barriers to cultural engagement later in life by creating opportunities for those in care settings and in community to have access to cultural teachings. Immersive, recurring education and training needs to be built in for staff to build the skills and reflexivity required for culturally congruent care, allowing for Indigenous knowledge to be integrated in care practices [34]. Feasibility challenges, such as funding for cultural rooms and the need for larger spaces to include families, require organizational commitment and policy alignment. Funding allocation must take a broader approach, focusing on community-oriented programs, including resources for home care, culturally congruent programs, care partner support, and staff education. The creation of an Indigenous-centered dementia care model must acknowledge the ongoing impacts of colonization and be prepared to not only shape care delivery but also act as a resistor to institutional pressures that could hinder implementation and uptake.

## 5. Conclusions

Though a broad approach was taken to recruitment, most of the participants were women. While deep insights were shared among all participants, this is a limitation that should be addressed by future research. This may have influenced the results and it will be important to evaluate the relevance of programming for persons living with dementia of all genders of any developed interventions based on this work. Though the findings stemmed from people from diverse Indigenous communities in Alberta, cultural distinctions may limit its transferability. However, given the limited work on understanding what is needed to create culturally congruent dementia care for Indigenous populations, this is an important starting point.

The aim of this research was to understand what is needed to create a culturally congruent dementia care model for Indigenous (First Nations, Métis, and Inuit) populations that can be adaptable depending on the context and culture. Specifically, because of the diversity of Indigenous cultures in urban settings, one community’s culture would not be able to be applied to one care facility, community care setting, and/or not-for-profit organization. It is important that this research be recognized for its aims and not to be a pan-Indigenized care model.

The understanding of the lived experience of dementia within Indigenous communities remains critically under-developed, creating a major gap in knowledge related to the diagnosis, treatment, and management of dementia for First Nations, Inuit, and Métis Peoples [1,5,7,11,35]. It is clear from the experiences bravely shared by each participant in this study that without action, unsafe care interactions will continue. Without the direction and leadership of Indigenous people with lived experiences in the research process, future supports and dementia care models risk overlooking critical factors to a culturally congruent and Indigenous-centered dementia care model.

## Figures and Tables

**Table 1:** Research participant demographics with pseudonyms.

Name	Gender	Indigenous Identity	Experience	Age (Years)	Sessions Attended
Alice	Woman	Métis	Health care worker in dementia care unit	36	4/4
Mary	Woman	Métis	Social work history, Care partner for parent	66	4/4
Dorothy	Woman	Métis/Cree/Dene	Care partner for Auntie	56	4/4
Joseph	Man	Métis/Cree	Care partner for multiple family members	38	4/4
Delores	Woman	Cree	Experiencing memory loss and was a care partner for spouse	67	4/4

**Table 2:** Details outlining the context of each focus group that took place.

Date	Location	Topic	Duration
6 October 2024	In-person—Edmonton Public Library	Relationality	90 min
5 November 2024	Online (ZOOM)	Being Well	72 min
28 November 2024	Online (ZOOM)	Safety	86 min
16 December 2024	Online (ZOOM)	Format	65 min

**Table 3:** Themes with the related subthemes and the relationship to descriptive codes.

Theme 1	Subthemes	Initial Codes
Indigenization	Family connection	Family at ceremony, community as family, equipping family as advocates
	Connection to culture	Cultural room, cultural teachings, cultural traditions in care, traditional food
	Understanding the context	Cultural safety, cultural admissions checklist, staff education
	Connecting back with the land	Access to nature, berry picking, green spaces
	Shared experiences	Physical spaces, relationality, sharing circles
Theme 2	Subthemes	Initial codes
Personalization	Connection to oneself	Individualized environments, brain stimulation, respecting individuality
	Reframing well-being	Mental health, strength-based questions, unique social needs
	Evolving accommodations	Accessibility for virtual gatherings, appointment accommodations, mobility strategies
	Autonomy	Food preferences, agency in care decisions, community calendar
	Diverse education opportunities	Lived experience learning opportunities, in-person and virtual accessibility
Theme 3	Subthemes	Initial codes
Intersection of Challenges and Innovations	Institutional reminders	Connection to residential school, intergenerational trauma
	Barrier to engage	Cross-cultural communication challenges, funding barriers, mistrust in current care
	Unsafe care stemming from racism	Presumptions by care staff, racism
	Accountability in implementation	Accreditation needs, staff education, staff retention, pan-Indigenization
	Mobilizing support	Collective efforts, advocacy and leadership, transit support

## Data Availability

Due to privacy and confidentiality and restrictions in ethical approval, raw data is unavailable.

## Supplementary Materials

The following supporting information can be downloaded at: https://www.mdpi.com/article/10.3390/ijerph23070883/s1, File S1: Focus Group Guide.
