# Supplementary material for: Indigenization, Personalization, and Intersections: A Qualitative Study to Identify Essential Components for Developing an Indigenous-Centered Dementia Care Model in Alberta
Source: Int J Environ Res Public Health. 2026 Jul 8;23(7):883. doi: 10.3390/ijerph23070883 (PMC13410778; doi:10.3390/ijerph23070883)
Supplement: Supplementary file 1 [file ijerph-23-00883-s001.zip › ijerph-4332231-supplementary.pdf]

Focus Group Guide

Focus Group 1: Guiding Framework-Relationality

1. **What are some ways to include community members in the care and support of individuals living with dementia?**
    a. What are some practical ways to do this?
    b. How do healthcare teams show that relationships are important in this type of care/what would this look like for you?
2. **What physical resources or spaces are most beneficial for fostering strong relationships in dementia care? (i.e., outdoor spaces, adaptive furniture, specific programs, etc.)**
    a. How might we design or modify physical spaces to better support these ideas?
    b. How might these resources and spaces need to change throughout disease progression?
3. **How can cultural practices and traditions be incorporated into dementia care to strengthen relational ties within the community?**
    a. What are some examples of traditional practices that you would like to see?

Focus Group 2: Guiding Framework- Being Well

1. **What social supports are essential for maintaining the well-being of Indigenous people living with dementia?**
    a. How might this change from what is needed at the time of diagnosis vs. later in disease?
    b. How might this change depending on where they are residing (e.g., home vs. care facility)?
2. **What physical barriers exist that prevent optimal well-being for Indigenous people living with dementia, and how can these be addressed?**
    a. Can you speak to that experience more?
    b. What does physical well-being look like for people living with dementia?
3. **What are some examples of how traditional practices have been used to promote well-being in your community?**
    a. What resources are necessary to support this?
    b. What are the current barriers?
    c. What do you think this could look like for Indigenous people living with dementia?

Focus Group 3: Guiding Framework-Safety

1. How can a care-model create safe social situations for Indigenous people living with dementia and **those** caring for them?
    a. What are the roles of family and community in ensuring the safety of Indigenous people living with dementia?
2. **What physical adaptations are necessary in homes and community spaces to ensure the safety of Indigenous people living with dementia?**
    a. How do these adaptations address current safety concerns?
3. **How can we ensure that health care workers are practicing culturally congruent care?**
    a. Probe: Can you speak more to that
    b. What does safety look like to you?

Focus Group 4: Format

1. **Length of program:**
    a. What would be an optimal length of each session?
    b. How many sessions should there be to effectively deliver Indigenous-centered dementia care?
2. **Delivery Method**:
    a. What are the advantages and things we would need to consider for conducting these sessions in person vs. virtually?
3. **Activities:**
    a. What types of activities should be included to engage participants and meet the goals of Indigenous-centered dementia care based on our previous discussions?
4. **Participants:**
    a. Who should attend these sessions to make sure we are taking a culturally congruent approach to dementia care?
